# Vestibular Migraine Patients Show Lack of Habituation in Auditory Middle Latency Responses to Repetitive Stimuli: Comparison With Meniere's Disease Patients

**DOI:** 10.3389/fneur.2020.00024

**Published:** 2020-02-20

**Authors:** Toshihisa Murofushi, Fumiyuki Goto, Masahito Tsubota

**Affiliations:** ^1^Department of Otolaryngology, Teikyo University School of Medicine Mizonokuchi Hospital, Kawasaki, Japan; ^2^Department of Otolaryngology, Tokai University School of Medicine, Isehara, Japan

**Keywords:** vertigo, auditory evoked potential, habituation, potentiation, sensory gating, mesencephalic reticular formation, thalamo-cortical pathway

## Abstract

**Objectives:** To compare habituation in auditory middle latency response (AMLR) to repetitive stimuli of vestibular migraine (VM) patients with Meniere's disease (MD) patients and healthy controls (HC) and to assess usefulness of AMLR for diagnosis of VM.

**Subjects:** Thirteen unilateral definite MD patients (2 men, 11 women, mean age 50.6), 13 definite VM patients (3 men, 10 women, mean age 45.5), and 8 HC subjects (2 men, 6 women, mean age 37.1) were enrolled.

**Methods:** The electrodes were placed on the vertex and the spinal process of the fifth cervical vertebra. Binaural click stimulation (0.1 ms, 70 dBnHL) was presented. A total of 800 responses were averaged. Averaged responses were divided into four sets (S1 to S4) according to the temporal order. No, Po, Na, and Pa were identified, and amplitudes and latencies were measured.

**Results:** Concerning latencies, HC subjects showed a tendency of shorter latencies. However, there was no clear effect of repetitive stimulation. Concerning No-Po amplitudes, no significant differences were observed. Raw amplitudes of Na-Pa showed statistically significant differences in S1 and S2 among the groups (*p* < 0.01 one-way ANOVA). Differences were shown in MD vs. VM and HC vs. VM in S1 (smaller in VM) (*p* < 0.01 Bonferroni's test) and in MD vs. VM in S2 (smaller in VM) (*p* < 0.01 Bonferroni test). Relative amplitudes of Na-Pa to S1 showed statistically significant differences in S4 (*p* < 0.01 one-way ANOVA). Differences were shown in MD vs. VM and HC vs. VM (larger in VM) (*p* < 0.01 Bonferroni's test). Differences of Na-Pa amplitudes in S2 to S4 from Na-Pa amplitude in S1 were significant in S4 of VM patients (Dunnett's test).

**Conclusions:** VM patients showed lack of habituation (potentiation) of Na-Pa amplitude in AMLR to repetitive stimuli while MD patients and HC subjects showed habituation. Observation of lack of habituation has high diagnostic accuracy for differential diagnosis of VM from MD.

## Introduction

Both Meniere's disease (MD) and vestibular migraine (VM) are representative diseases that are presented with episodic vertigo attacks lasting for several hours (1). Recently, diagnostic criteria of MD were revised and those of VM were established (2, 3). Their clinical diagnoses basically depend on medical history. Differentiation of the two diseases is sometimes hard. Therefore, objective tests are carried out to help clinicians differentiate the two diseases. In other words, biomarkers with neurophysiological methods for differential diagnosis of the two diseases are required.

As endolymphatic hydrops (EH) is one of the histopathological features of MD, clinical tests for detection of EH such as cervical vestibular evoked myogenic potential (cVEMP), electrocochleography (ECoG), and so on may provide useful information (4–7). Nowadays, MRI has also been applied for detection of EH (8, 9) On the other hand, clinical tests suggestive of VM are not established. It has been known that patients with migraine show some neurophysiological features in interictal periods. They are (a) lack of habituation to repetitive stimuli (10–12), (b) thalamocortical dysrhythmia (11), and (c) dysfunction of the descending pathways of pain modulation (13). Among them, we paid attention to lack of habituation to repetitive stimuli in migraine patients.

Lack of habituation to repetitive stimuli in migraine patients was shown using visually evoked potential (VEP) (10, 11), auditory slow vertex response (SVR), and so on (10, 14). Here, “habituation” implies a response decrement as a result of repeated stimulation (15). However, differences of auditory evoked responses to repetitive stimuli between VM patients and MD patients are not known, although differential diagnosis of these two diseases is sometimes difficult. In this study, we tried to show differences of habituation (or potentiation) in the auditory middle latency response (AMLR) (16, 17), which is one of the auditory evoked potentials in the central nervous system, between VM and MD. If the response patterns to repetitive stimuli are clearly different, observation of the difference might be applicable as a clinical test for differential diagnosis of the two diseases.

## Materials and Methods

### Subjects

Thirteen definite VM patients (3 men and 10 women, mean age 45.5, age range 30–69), 13 unilateral definite MD patients (2 men and 11 women, mean age 50.6, age range 31–79), and 8 healthy control (HC) subjects (2 men and 6 women, mean age 37.1, age range 24–50) were recruited and enrolled into this study. The affected sides of MD were right in 7 patients and left in 6 patients. Patients with vestibular migraine/Meniere's disease overlapping syndrome (VM/MD-OS) (18) were not included.

Recording of VM and MD patients were performed in the interictal period. Duration after the last attack was diverse.

### Methods

#### Recording of AMLR

AMLRs were recorded using Neuropack system (Nihon Kohden Co. Ltd., Japan), The active electrode was placed on the vertex, while the inactive electrode was on the spinal process of the fifth cervical vertebra. The ground electrode was on the nasion. The subjects were awake with the eyes closed in the supine position. Click stimulation (0.1 ms, 70 dBnHL) was binaurally presented through the headphone (Elga Acous. Co. Ltd., Japan). Repetition rate was 5 Hz. Signals were bandpass-filtered (20–1,000 Hz) and a total of 800 responses were averaged. Averaged responses were divided into four sets. Set 1 (S1) was averaging of the first 200 responses; Set 2 (S2), the second 200 responses; Set 3 (S3), the third 200 responses; and Set 4 (S4), the fourth 200 responses. Recording was performed by the authors.

#### Nomenclature

**Figure 1:**
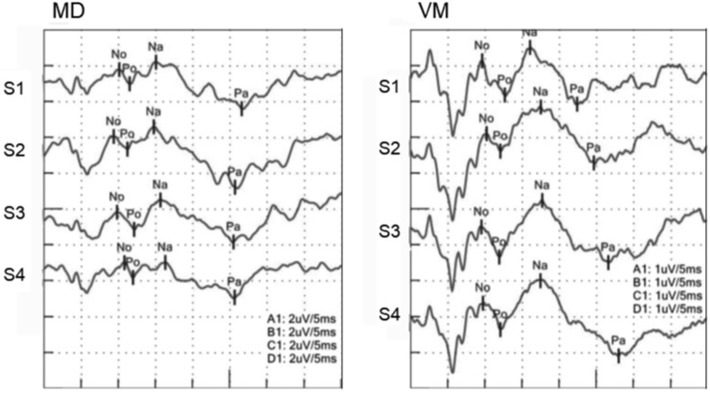
AMLRs to repetitive stimuli. **(Left)** AMLR of an MD patient. **(Right)** AMLR of a VM patient. Although an MD patient showed decreasing Na-Pa amplitudes to repetitive stimuli, a VM patient showed increasing Na-Pa amplitudes to repetitive stimuli.

Nomenclature of Picton et al. was adopted (Figure 1) (17). Then No, Po, Na, and Pa were named as peaks of responses. No was defined as the largest negative deflection that followed wave 5 of auditory brainstem response (ABR) and was earlier than 15 ms. Po was defined as the largest positive deflection which followed No and was earlier than 20 ms. When we did not find such peaks, we did not name No or Po. Na was defined as the largest negative deflection that followed Po and was earlier than 25 ms. When No was not found, the largest negative deflection which followed wave 5 and was earlier than 25 ms was regarded as Na. Pa was defined as the largest positive deflection that followed Na and earlier than 40 ms. When we could not decide Po or Na, the largest positive deflection earlier than 40 ms was defined as Pa. Peak-to-peak amplitudes of No-Po and Na-Pa and peak latencies of No, Po, Na, and Pa were measured. Identification of the peaks was performed by the authors.

#### Comparison of Habituation Among the Three Groups

In order to examine habituation to repetitive stimuli, raw amplitudes of No-Po and Na-Pa and relative amplitudes to those of Set 1 were measured and compared. Latencies of No, Po, Na, and Pa to the onset of stimulation were also measured and compared.

#### Statistical Analyses

For statistical analyses, one-way ANOVA was used. For multiple comparison, Bonferroni's test and Dunnett's test were used. *p* < 0.05 was regarded as significant.

Informed consent was obtained from each subject. This study was approved by the Ethics Committee of Teikyo University (TR15-021). This study was performed in accordance with Declaration of Helsinki (1964) and its later amendments.

## Results

### Age and Hearing Level

The mean ages of the three groups (MD = 50.6 ± 13.6, VM = 45.5 ± 13.7, HC = 37.1 ± 9.87, mean ± SD years of age) had no significant difference (*p* > 0.05, one-way ANOVA). The mean hearing levels of the four groups (MD-right ear = 20.7 ± 13.5, MD-left ear = 20.6 ± 19.6, VM-right ear = 16.9 ± 8.6, VM-left ear = 15.0 ± 9.1, mean ± SD dBHL) showed no significant difference (*p* > 0.05, one-way ANOVA).

### Identification of Peaks

In one VM patient, we could not identify No and Po. In one MD patient, we could not identify Po and Na. In one HC subject, we could not identify Po and No. Other subjects showed all the peaks. Therefore, we could not measure No-Po amplitudes in one VM patient and one healthy subject, and either of No-Po or Na-Pa amplitudes in one MD patient.

### No-Po Amplitude

Concerning No-Po amplitudes, no clear amplitude changes to repetitive stimuli were observed. There was no significant difference among the three groups in either of raw amplitudes or relative amplitudes to Set 1 (*p* > 0.05, one-way ANOVA) (Table 1).

**Table 1:** Amplitudes of No-Po.

	***N***	**S1**	**S2**	**S3**	**S4**
**Raw amplitudes (mean** **±** **SE** **μV)**					
MD	12	0.74 ± 0.092	0.71 ± 0.17	0.86 ± 0.12	0.61 ± 0.13
VM	12	0.74 ± 0.082	0.71 ± 0.089	0.80 ± 0.14	0.64 ± 0.098
HC	7	1.14 ± 0.29	1.14 ± 0.30	0.88 ± 0.15	0.85 ± 0.27
*p*-value		*p* > 0.05	*p* > 0.05	*p* > 0.05	*p* > 0.05
	***N***	**S2**	**S3**	**S4**	
**Relative amplitudes to S1 (mean** **±** **SE)**					
MD	12	0.98 ± 0.20	1.03 ± 0.17	0.81 ± 0.12	
VM	12	1.13 ± 0.22	1.06 ± 0.26	0.93 ± 0.14	
HC	7	1.28 ± 0.35	1.22 ± 0.39	1.16 ± 0.34	
*p*-value		*p* > 0.05	*p* > 0.05	*p* > 0.05	

*There was no significant difference among the groups*.

### Na-Pa Amplitude

MD patients and HC subjects showed tendency of declining of Na-Pa amplitudes in Sets 3 and 4. On the other hand, VM patients showed potentiation (lack of habituation) of Na-Pa amplitudes after repetitive stimulation.

Raw amplitudes of Na-Pa showed statistically significant differences in S1 and S2 among the groups (*p* < 0.001 in S1 and *p* = 0.002 in S2, one-way ANOVA). Differences were shown in MD vs. VM and HC vs. VM in S1 and S2 (Bonferroni's test, Table 2). Differences of Na-Pa amplitudes in S2 to S4 from Na-Pa amplitude in S1 were significant only in S4 of VM patients (Dunnett's test, Table 2).

**Table 2:** Amplitudes of Na-Pa.

	***N***	**S1**	**S2**	**S3**	**S4**
**Raw amplitudes (mean** **±** **SE** **μV)**					
MD	12	2.22 ± 0.23	2.24 ± 0.23	2.04 ± 0.15	1.63 ± 0.15
VM	13	1.41 ± 0.11	1.63 ± 0.11	1.74 ± 0.20	1.89 ± 0.12*
HC	8	2.54 ± 0.20	2.56 ± 0.19	2.41 ± 0.32	1.99 ± 0.24
*p*-value		*p* < 0.001	*p* = 0.002	NS	NS
S1: MD vs. VM *p* = 0.0089, MD vs. HC *p* > 0.05, VM vs. HC *p* < 0.001 Bonferroni's test					
S2: MD vs. VM *p* > 0.05, MD vs. HC *p* > 0.05, VM vs. HC *p* = 0.0061 Bonferroni's test					
*S4 of VM was significantly larger than S1 of VM (*p* = 0.030, Dunnett's test)					
	***N***	**S2**	**S3**	**S4**	
**Relative amplitudes to S1 (mean** **±** **SE)**					
MD	12	1.10 ± 0.13	1.01 ± 0.08	0.78 ± 0.08	
VM	13	1.22 ± 0.11	1.28 ± 0.15	1.40 ± 0.31	
HC	8	1.04 ± 0.11	0.928 ± 0.078	0.82 ± 0.11	
*p*-value		*p* > 0.05	*p* > 0.05	*p* = 0.002	
S4: MD vs. VM *p* < 0.001, MD vs. HC *p* > 0.05, VM vs. HC *p* = 0.005 Bonferroni's test					

*Raw amplitudes of VM patients in S1 and S2 showed significantly smaller amplitudes than other groups (one-way ANOVA). Relative amplitude of S4 to S1 of VM patients was significantly larger than other groups (one-way ANOVA)*.

**Figure 2:**
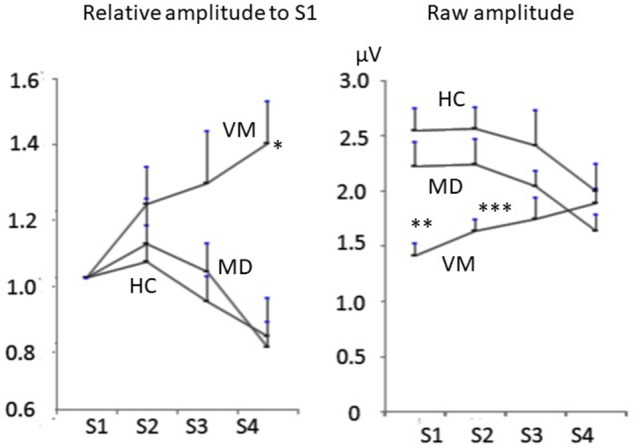
Change of Na-Pa amplitudes to repetitive stimuli. **(Left)** relative amplitudes to S1. **(Right)** raw amplitudes. Graphs represent mean and SE. *p = 00.002, **p < 0.001, ***p = 0.002 one-way ANOVA.

Relative amplitudes of Na-Pa to S1 showed statistically significant differences in S4 (*p* = 0.002, one-way ANOVA). Differences were shown in MD vs. VM and HC vs. VM (Bonferroni's test) (Figures 1, 2, Table 2).

**Figure 3:**
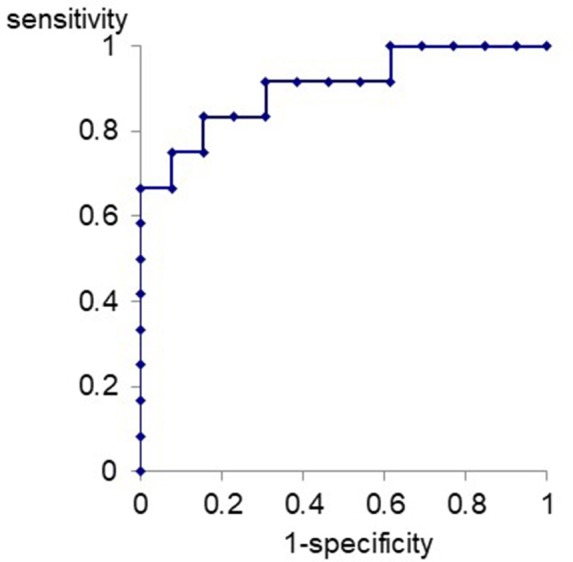
ROC curve for differentiation of VM patients from MD patients.

ROC curve for differentiation of VM patients from MD patients with the relative amplitude of Na-Pa in S4 to S1 was produced (Figure 3). The best cutoff line for differentiation of VM patients from MD patients using the relative Na-Pa amplitude in S4 to S1 was 1.11. Then, sensitivity, specificity, and AUC (area under the curve) were 0.83, 0.84, and 0.90, respectively.

### Latencies

Although HC group had tendency of shorter latencies than the MD and VM groups, no clear effects of repetitive stimulation were not observed (Table 3).

**Table 3:** Latencies (mean ± SE ms).

	***N***	**S1**	**S2**	**S3**	**S4**
**No**					
MD	13	9.85 ± 0.38	9.68 ± 0.38	10.00 ± 0.35	9.86 ± 0.36
VM	12	9.33 ± 0.28	9.01 ± 0.25	9.10 ± 0.41	9.55 ± 0.16
HC	7	8.46 ± 0.22	8.40 ± 1.4	9.17 ± 0.38	8.55 ± 0.39
*p*-value		*p* = 0.01	*p* > 0.05	*p* > 0.05	*p* > 0.05
S1: MD vs. HC *p* = 0.04, MD vs. VM and VM vs. HC *p* > 0.05 Bonferroni's test					
**Po**					
MD	12	13.1 ± 0.37	12.9 ± 0.58	13.1 ± 0.36	13.6 ± 0.44
VM	12	13.3 ± 0.49	12.1 ± 0.36	13.9 ± 0.56	13.0 ± 0.32
HC	7	11.6 ± 0.48	12.5 ± 0.26	12.0 ± 0.37	11.9 ± 0.38
*p*-value		*p* > 0.05	*p* > 0.05	*p* > 0.05	*p* = 0.03
S4: MD vs. HC *p* = 0.02, MD vs. VM and VM vs. HC *p* > 0.05 Bonferroni's test					
**Na**					
MD	12	16.9 ± 0.59	16.9 ± 0.49	17.1 ± 0.47	17.5 ± 0.39
VM	13	16.6 ± 0.42	16.3 ± 0.32	16.8 ± 0.44.	17.3 ± 0.42
HC	7	16.7 ± 0.48	16.4 ± 0.48	16.7 ± 0.67	16.4 ± 0.71
*p*-value		*p* > 0.05	*p* > 0.05	*p* > 0.05	*p* > 0.05
**Pa**					
MD	13	27.7 ± 0.48	28.0 ± 0.62	27.7 ± 0.68	25.5 ± 1.65
VM	13	26.5 ± 0.88	27.5 ± 0.71	28.4 ± 0.70	28.6 ± 0.91
HC	7	26.3 ± 0.87	27.7 ± 1.1	25.7 ± 0.58	22.7 ± 3.37
*p*-value		*p* > 0.05	*p* > 0.05	*p* = 0.02	*p* > 0.05
S3: MD vs. HC, MD vs. VM, and VM vs. HC *p* > 0.05					

*Although HC group had tendency of shorter latencies than the MD and VM groups, no clear effects of repetitive stimulation were observed*.

## Discussion

The present study showed that VM patients showed lack of habituation or potentiation to repetitive stimuli in AMLR although the study was relatively small-sized. Lack of habituation, or potentiation to repetitive stimuli has been reported in various evoked potentials of migraine patients (10–12, 14). Wang et al. recorded SVR using 1,000 Hz tone bursts in migraine patients and controls, reporting migraine patients showed potentiation of responses in Sets 2 and 3 to Set 1 to 70 dB SL while control subjects showed habituation (19). Ambrosini et al. also reported potentiation of N1-P2 amplitudes of SVR (14). As a feature in AMLR of migraine patients, Ambrosini et al. showed that the P50 amplitude of migraine patients to the second stimulation in paired-stimuli had less decrease than that of HC (20). P50 in their study seems to correspond to Pb in the nomenclature by Picton et al. (17). Ambrosini et al. speculated that this phenomenon might be caused by impaired auditory “sensory gating” (20). Sensory gating, a central phenomenon that plays an important role in the processing of incoming information, causes suppression of the cortical response. This process allows the central nervous system to pay an attention selectively to important stimuli while ignoring redundant stimuli (21). Habituation to repetitive stimuli and sensory gating might reflect similar neurophysiological phenomena. Lack of habituation and impaired sensory gating might be attributed to hypofunction of the raphe nuclei in the mesencephalic reticular formation (20).

In the present study, we first reported potentiation of Na-Pa amplitude to repetitive stimuli in AMLR of VM patients and that this phenomenon in VM patients was not observed in MD patients, although both diseases show similar episodic vertigo attacks. AUC of ROC curve concerning differentiation of VM patients from MD patients using the relative Na-Pa amplitude in S4 to S1 was 0.90. It means differentiation of VM from MD using this parameter has high diagnostic accuracy (22).

As MD patients have sensorineural hearing loss to some extents, effects of hearing loss have to be considered. In this study, we only enrolled unilateral MD patients and presented binaural auditory stimulation. Comparison of mean hearing levels of the MD-right ear, MD-left ear, VM-right ear, and VM-left ear did not show significant difference. Therefore, we could minimize the effects of hearing loss. Furthermore, results in HC subjects supported that lack of habituation in AMLR in VM patients is attributable to disorders in the central processing. In this study, recording was performed in the interictal period for both vertigo attacks and headache attacks. Therefore, we cannot tell relationships between AMLR findings and duration after attacks.

In this study, we measured AMLR at the midline. The electrodes were placed on the vertex and the spinal process of the fifth cervical vertebra. We adopted this midline montage for two reasons. Firstly, because we presented clicks binaurally in order to minimize effects of hearing loss in MD patients, recording was done in the midline. Secondly, we adopted this montage to avoid contamination of myogenic responses such as post-auricular myogenic responses (23).

Pathophysiology of habituation disorders in migraine patients is not fully understood. According to de Tommaso et al. (11), habituation deficits could be caused by (a) increased excitatory mechanism, (b) decreased activity of inhibitory interneurons, or (c) reduced baseline activation of sensory cortices with the “ceiling theory.” The ceiling theory in lack of habituation of migraine patients is based on the hypotheses that sensory cortices have variable baseline activation levels but constant maximum activation levels and that migraine patients have low baseline activation. Findings of the present study is consistent with the ceiling theory because Na-Pa amplitudes in VM patients were initially lower than MD patients and HC subjects and they were increased by repetitive stimulation. If hypothesis (a) or (b) was correct, the initial responses in VM patients should be the same as MD patients and HC subjects or larger.

As generators of waves in AMLR, several areas have been proposed. As generators of wave Pa by the midline montage, the thalamocortical pathway and mesencephalic reticular formation as well as the primary auditory cortex are supposed (16, 24, 25). For wave Na by the midline montage, the midbrain plays an important role (16). Although generators of waves No and Po are poorly explored, they should be generated in the midbrain because wave 5 of ABR, which precedes wave No, is generated around the inferior colliculus (26). Therefore, the present study suggested that potentiation of Na-Pa amplitude to repetitive stimuli in AMLR of VM patients by the midline montage could be associated not only with the primary auditory cortex but also with the thalamocortical pathway and mesencephalic reticular formation. In combination with the hypothesis of impaired sensory gating, potentiation of Na-Pa amplitude in AMLR of VM patients to repetitive stimuli in the present study might be attributed to dysfunction of the raphe nuclei, a part of the mesencephalic reticular formation (20).

In conclusion, lack of habituation (or potentiation) of Na-Pa amplitude in AMLR of VM patients to repetitive stimuli was first confirmed. This response pattern was totally different from that of MD patients. For lack of habituation (or potentiation) observed in the present study, dysfunction of the raphe nuclei, a part of the mesencephalic reticular formation, as well as the thalamocortical pathway and primary auditory cortex might play an important role. Observation of lack of habituation (or potentiation) in AMLR of VM patients might be helpful for differential diagnosis of VM from MD. The possibility of differential diagnosis between VM and migraine without vertigo remains to be clarified in the future.

## Data Availability Statement

All datasets generated for this study are included in the article/supplementary material.

## Ethics Statement

The studies involving human participants were reviewed and approved by the Ethics Committee of Teikyo University. The patients/participants provided their written informed consent to participate in this study.

## Author Contributions

All authors contributed extensively to the work presented in this paper. All authors collected data. TM wrote the manuscript. MT and FG reviewed and edited the manuscript.

### Conflict of Interest

The authors declare that the research was conducted in the absence of any commercial or financial relationships that could be construed as a potential conflict of interest.
